# Syphilis-associated septic cardiomyopathy: case report and review of the literature

**DOI:** 10.1186/s12879-020-05722-z

**Published:** 2021-01-07

**Authors:** Shiqi Guo, Qiang Guo

**Affiliations:** 1 Department of Pulmonary and Critical Care Medicine, The First Affiliated Hospital of Soochow University, Suzhou, Jiangsu China; 2 Department of Emergency, Suzhou Dushuhu Public Hospital (Dushuhu Public Hospital Affiliated to Soochow University), No.9 Chongwen Road, Suzhou Industrial Park, Suzhou, 215000 Jiangsu China; 3 Department of Pulmonary and Critical Care Medicine, Suzhou Dushuhu Public Hospital (Dushuhu Public Hospital Affiliated to Soochow University), Suzhou, Jiangsu China

**Keywords:** Case report, Septic cardiomyopathy, Sepsis-induced cardiomyopathy, Syphilis, Treponema pallidum

## Abstract

**Background:**

Septic cardiomyopathy has been observed in association with influenza, indicating that not only bacteria but also other infective agents can cause this condition. There has been no systematic study as to whether Treponema pallidum infection induces septic cardiomyopathy, and we are the first to report this possibility.

**Case presentation:**

We report two cases of a 48-year-old man and a 57-year-old man who were diagnosed with syphilis-related septic cardiomyopathy. The diagnosis of cardiomyopathy was made based on elevation of cardiogenic markers and decrease in ejection fraction evaluated by echocardiography. Screen for infective pathogens was negative except for syphilis, which supported our diagnosis. The two patients recovered following effective anti-syphilis treatment and advanced life support technology. Syphilis serology became negative after treatment.

**Conclusion:**

Syphilis has the potential to cause septic cardiomyopathy. Clinicians should consider Treponema pallidum in cases of septic cardiomyopathy with unknown pathogens. However, the specific pathophysiological mechanism of syphilis-associated septic cardiomyopathy has not been elucidated, and more specific studies are needed.

## Background

Based on the current international consensus definition, sepsis is a life-threatening organ dysfunction caused by the host’s response to infection [1, 2]. However, the definition of septic cardiomyopathy (SC) remains controversial; an acute disturbance of cardiac function in patients with sepsis is generally evaluated as SC [3]. The diagnosis is made mainly based on echocardiographic findings, including left ventricular ejection fraction (EF) < 0.5, decreased left ventricular longitudinal strain, and increased left ventricular end-diastolic diameter. Continuous changes in markers of myocardial damage, especially troponin and N-terminal pro-brain natriuretic peptide (NT-proBNP) reflect the degree of myocardial injury [3, 4], and these are thus helpful for the diagnosis of myocardial cell injury in sepsis. In addition, the syndrome of septic cardiomyopathy is usually reversible, with patients gradually recovering within 7–10 days [4, 5]. A diagnosis of SC is thus considered in the presence of increased inflammatory indicators and dysfunction in the coagulation, liver, kidneys, or lungs, combined with EF < 0.5 and increased myocardial enzymes, after excluding coronary artery disease.

Septic cardiomyopathy has been reported in association with influenza [4], indicating that not only bacteria but also other infective agents can cause SC. Here we describe two cases of septic cardiomyopathy associated with syphilis. We searched PubMed with the following terms “septic cardiomyopathy + syphilis” but did not find relevant research or report and thus, to our knowledge, this is the first case report of this association.

## Cases presentation

### Case 1

**Fig. 1:**
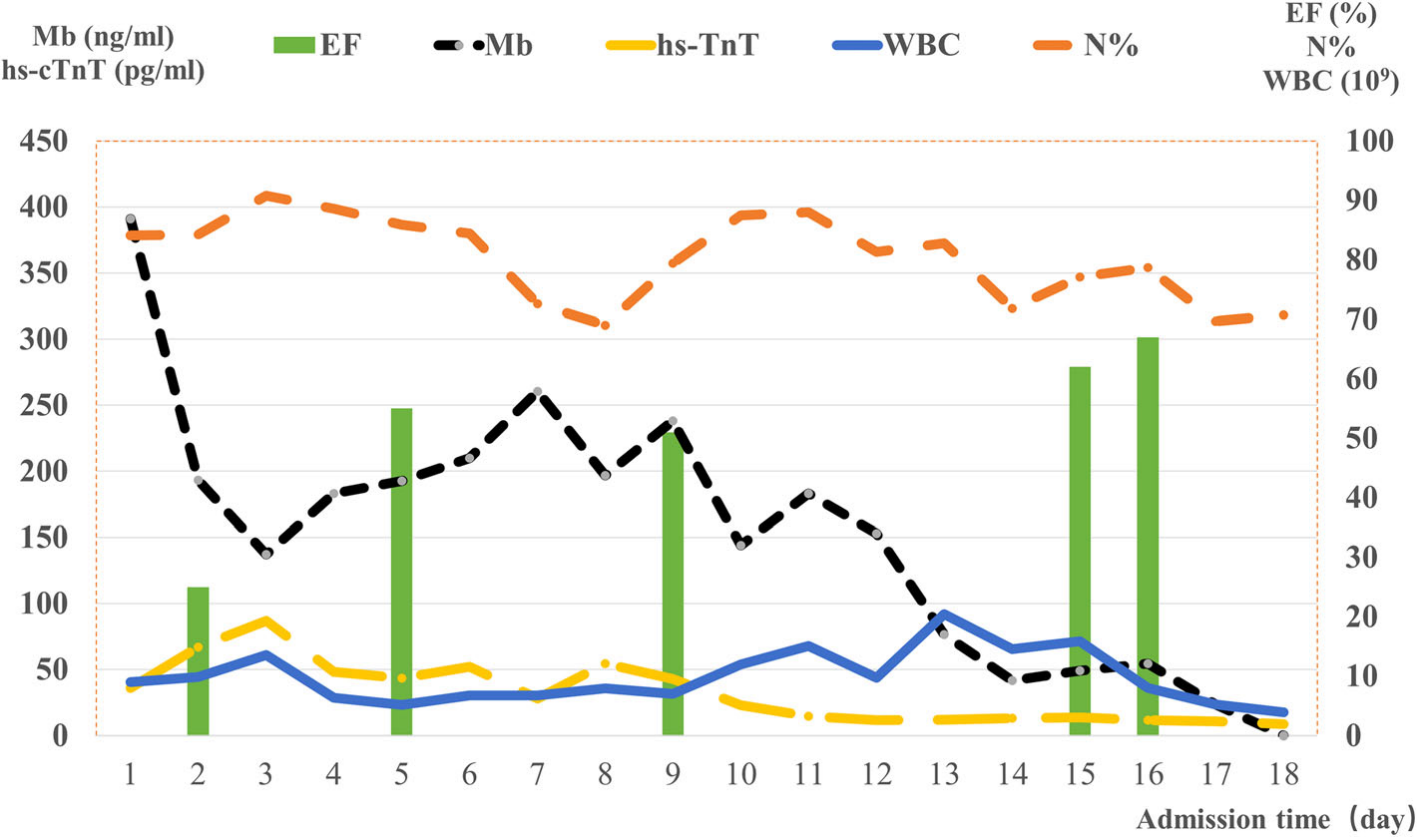
Clinical course of the case of a 48-year-old man. Cardiogenic markers and inflammatory indicators gradually decreased. Echocardiography indicated that the EF returned to normal within 10 days. EF: ejection fraction

**Fig. 2:**
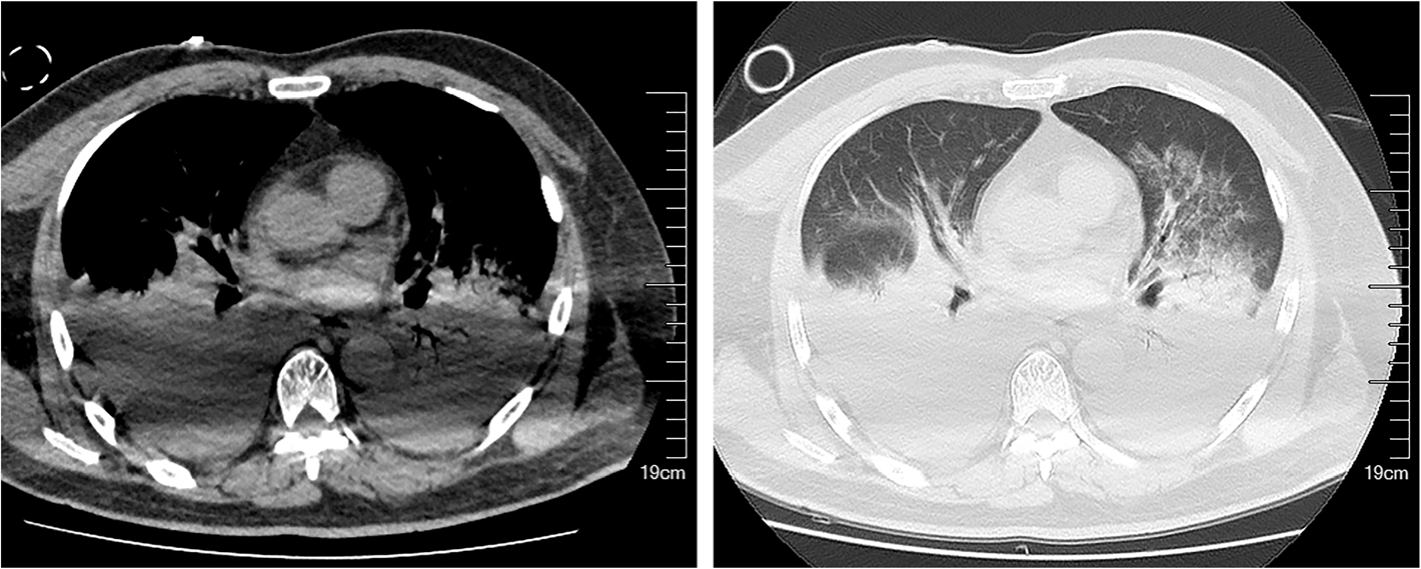
Chest CT scan in case 1 at admission. Bilateral clear lung fields and patchy high-density shadows in the lobi inferior indicated bilateral pneumonia and pulmonary edema. The shape and diameter of the heart and great blood vessels were normal, with a small amount of pericardial effusion and large bilateral pleural effusions. CT: computed tomography

A 48-year-old man was hospitalized on November 1, 2019, due to recurrent fever for more than a month after a business trip. The patient developed fever accompanied by rigors. His body temperature was 40 °C, and he complained of shortness of breath, chest tightness, wheezing, dizziness, and fatigue, accompanied by scattered purpura. Blood pressure was measured at 55/40 mmHg. The electrocardiogram showed sinus rhythm with a heart rate of 50 beats/min, and no ST segment changes were detected. Laboratory tests showed the following: white blood cell count (WBC) 8.97× 10^9^/L (reference range 3.5–9.5× 10^9^/L), neutrophil percentage (N%) 84.10 (reference range 40.0–75.0); C-reactive protein (CRP), 94.4 mg/L (reference range 0–8 mg/L); procalcitonin, 3.86 μg/L (reference range 0–0.5 g/L); hypersensitive troponin T (hs-cTnT) 36.13 pg/mL (reference range 0–14 pg/mL); myoglobin (Mb) 391 ng/mL (reference range 0–72 ng/mL); pH 7.387 (reference range 7.35–7.45); PCO_2_ 18.5 mmHg (reference range, 32–48 mmHg); lactate 2.3 mmol/L (reference range 0.5–1.6. mmol/L) (continuous changes in some indicators are presented in Fig. 1). Cardiac insufficiency was detected by bedside echocardiography with the left ventricular wall motion significantly weakened with an EF of 0.25. Normal valves and slight tricuspid regurgitation during systole were revealed. Septic cardiomyopathy was thus diagnosed. On day 1, a chest computed tomography (CT) scan (Fig. 2) showed patchy high-density shadows in the lobi inferior, suggesting bilateral pneumonia and pulmonary edema. The shape and diameter of the heart and great blood vessels were normal, with a small amount of pericardial effusion and large bilateral pleural effusions. After obtaining pathogenic specimens, we administered imipenem as broad-spectrum antibiotic cover before the test results were available. We routinely screen inpatients for human immunodeficiency virus (HIV), syphilis, hepatitis A, B, C, and other infectious diseases, and found a Treponema pallidum particle agglutination (TPPA) titer of 1:80 in this patient. He reported previous venereal exposure and recalled three irregular intramuscular injections for treatment of genital ulcers (not found this time) 6 months ago, but he defaulted treatment on his own and did not have any further review to monitor the progression of the syphilis. Toluidine red unheated serum test (TRUST) was positive, and the rapid plasma reagin (RPR) test titer was 1:64, indicating active early syphilis. Based on the results, we continued to use carbapenems to treat syphilis (imipenem 3.0 g/day) continuously for 14 days. Other treatments such as continuous renal replacement therapy (CRRT) and invasive mechanical ventilation (IMV) were required within 24 h. On day 2, due to the continuous decrease in oxygenation, the catheters for extracorporeal membrane oxygenation (ECMO) were inserted. We took samples of body fluids for culture on admission, including blood, sputum, and pleural effusion, which showed aseptic growth on day 5. The results of fungal tests and bronchoalveolar lavage (BAL) for next-generation sequencing technology (Ngs) (DNA and RNA) prior to commencement of antibiotics were negative. Bedside echocardiography on day 5 indicated ongoing mild tricuspid regurgitation, but there was no obvious abnormality in ventricular wall motion, and the left ventricle had an inner diameter of 32 mm at the end of systole, with EF of 0.55. On day 6, the patient was free from ECMO. On day 13, he received high-flow nasal cannula oxygen therapy instead of IMV. On the same day, the CRRT and hemodialysis catheter was removed because the urine volume had increased and the creatinine clearance rate had returned to normal. Cardiogenic markers and inflammatory indicators gradually decreased. Mb, and hs-cTnT returned to normal shortly thereafter. On day 16, the patient’s vital signs were stable. Repeated echocardiography showed no obvious abnormalities in ventricular wall motion. There was no obvious abnormality in the morphology or movement of each valve or any obvious regurgitation. The inner diameter of the aortic root was 37 mm. In addition, re-examination of RPR showed a gradual decrease in antibody titer (1:2), and TRUST finally became negative. Therefore, syphilis-associated SC was diagnosed.

### Case 2

**Fig. 3:**
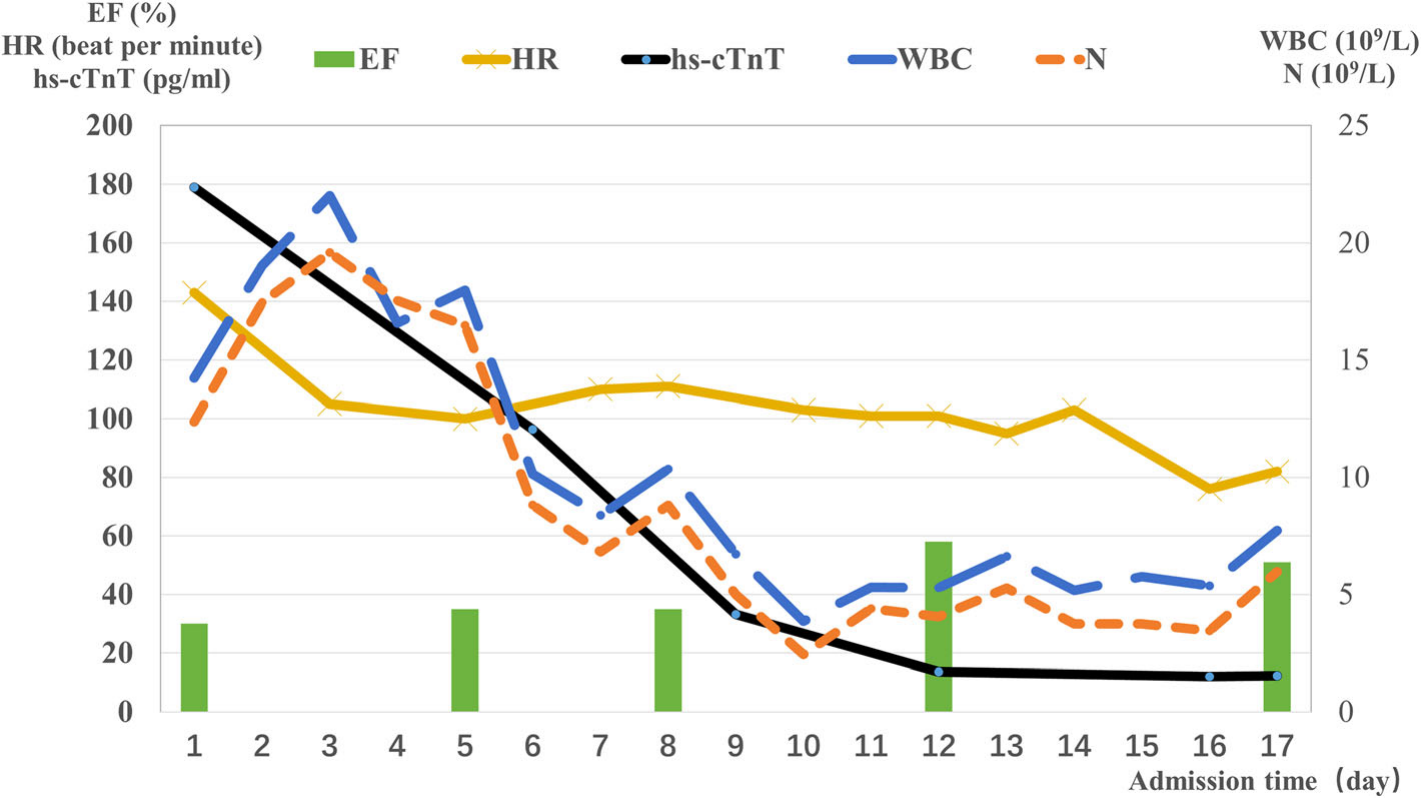
The Clinical course of the case of a 51-year-old man. Inflammatory indicators and cardiogenic markers gradually decreased and returned to normal after 10 days. Echocardiography indicated that the EF returned to normal within two weeks

The second patient was a 57-year-old man hospitalized on April 30, 2020, due to repeated tightness in the chest and abdominal distension for three days, along with a high fever with a maximum body temperature of 40.3 °C. Laboratory tests showed a marked increase in inflammatory indices: CRP > 15.36 mg/L; WBC 14.23× 10^9^; neutrophil count (N) 12.37× 10^9^ (reference range: 1.8–6.3× 10^9^); N% 86.9; PH 7.317; PaO_2_/FiO_2_ 231 mmHg; hs-cTnT 179.0 pg/mL; NT-proBNP 2049 pg/mL (reference range: 0–125 pg/mL) (continuous changes of some indicators are presented in Fig. 3).

**Fig. 4:**
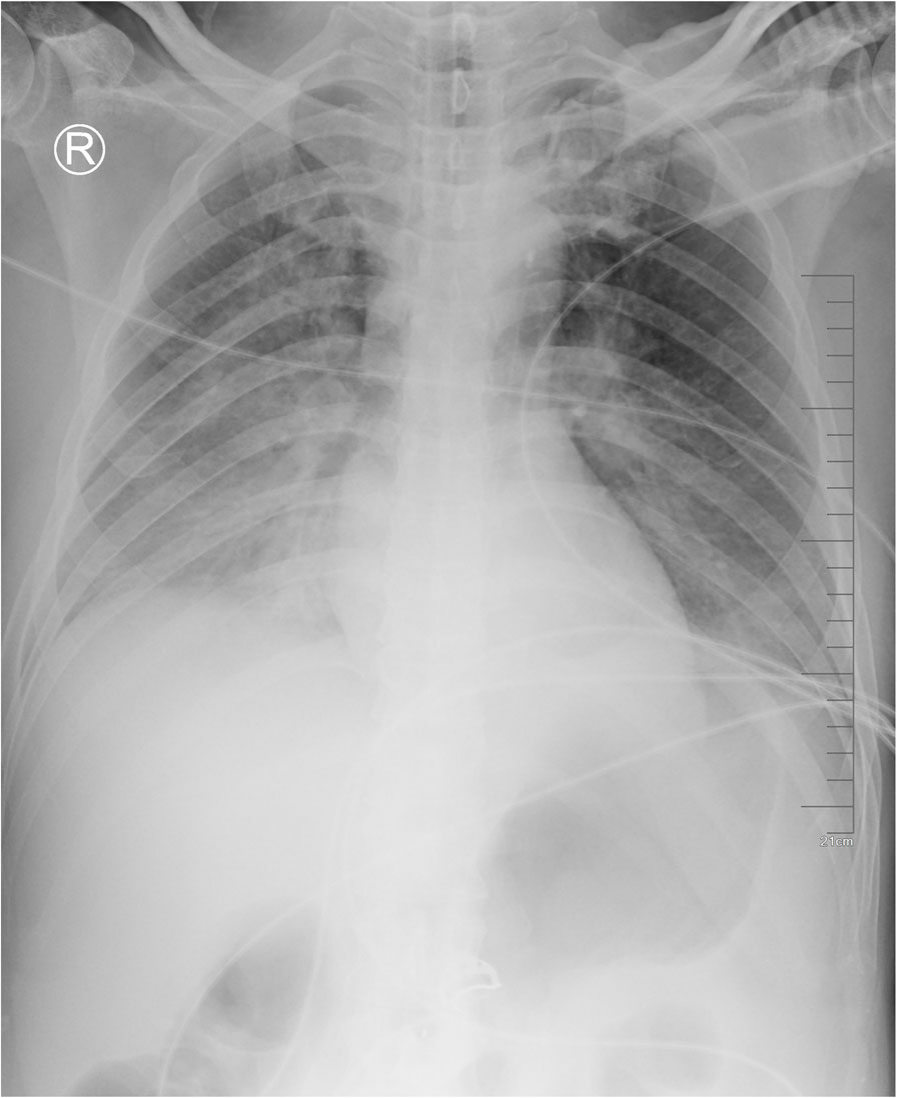
Chest x-ray findings in case 2 at admission. The texture of both lungs was clear; the light transmittance was reduced, and patchy fuzzy shadows were observed bilaterally, whereas the shape and size of the heart shadow were normal

No apparent changes were detected on electrocardiography. Echocardiography suggested a left ventricular end-systolic diameter of 35 mm with an EF of only 0.3. Moreover, the motion of the anterior wall of the left ventricle and the apex of the ventricle were weakened, and the apex of the left ventricle bulged out slightly during systole. The morphology and activity of each valve were normal, and the aortic valve had slight regurgitation during diastole. Chest radiography (Fig. 4) revealed that the texture of the both lungs was clear; the light transmittance was reduced, and patchy fuzzy shadows were observed bilaterally, while the shape and size of the heart shadow were normal. The patient was administered imipenem 2.0 g/day, intravenously every 6 h, continuously for 14 days. Screening for infections, including SARS-CoV-2, was negative except for a TPPA titer of 1:80, RPR titer of 1:64, and TRUST positive, revealing that the patient had active syphilis. He denied the primary infection of syphilis and could not accurately recall whether genital ulcers had occurred; none were observed at this presentation. He did admit to having unprotected sex several times in the past year. Due to a lack of history of previous testing or treatment, but no imaging manifestations of cardiovascular syphilis, such as aortic widening and linear calcification, early syphilis was considered. Further supportive treatments such as IMV, dual antiplatelet therapy, inhibiting ventricular reconstruction, and intra-aortic balloon pump (IABP), which continued to work with a counter pulsation frequency of 1:1, were used. On day 5, the results of blood and sputum cultures on admission suggested aseptic growth, and BAL for Ngs (DNA and RNA) was negative. At repeat echocardiography, we found that the patient’s EF was only 0.35. The anterior wall of the left ventricle and the apical motion of the heart was diminished, and the aortic valve had slight regurgitation in diastole. On day 8, the circulation gradually stabilized, so the counter pulsation frequency of IABP was decreased to 1:2. On day 12, after systematic and effective treatment, inflammatory indicators and cardiogenic markers, such as hs-cTnT, gradually decreased and finally returned to normal. In addition, bedside echocardiography demonstrated a normal range of ventricular wall motion at rest and normal shape and movement of each valve, although slight aortic regurgitation during diastole persisted. As the patient’s cardiac function was acceptable and the EF was 0.58, the IABP was successfully removed. The re-examination of blood gas after trying offline showed an oxygenation index of 432 mmHg, and the ventilator was thus removed on the same day. Norepinephrine was stopped 3 days after discontinuing the invasive respiratory ventilation. On day 15, RPR showed that the titer of the non-specific antibody had dropped to 1:2 and TRUST finally became negative. Therefore, SC caused by syphilis infection was diagnosed.

## Discussion and conclusion

SC manifested in two patients with severe infection accompanied by multiple organ dysfunction, elevated myocardial markers, and decreased EF values. The diagnosis of syphilis-associated SC emphasized three important clinical insights: (I) Routine etiologic examination results of these patients were negative, and only Treponema pallidum antibody was positive. (II) We ruled out other cardiac disorders. There were no ST segment changes on electrocardiography, thus excluding coronary atherosclerotic heart disease and other serious cardiac arrhythmias. Neither patient had a history of hypertension, and thus hypertensive heart disease was not considered. They were both in the early stages of syphilis, and no evidence of cardiovascular syphilis was found. (III) After treatment, the structure and function of the heart were fully restored to normal within 2 weeks.

We sent specimens for examination before commencing antibiotics, and used broad-spectrum antibiotics empirically before obtaining results; imipenem was administered to both patients. Initial tests were positive for syphilis, and we continued the use of imipenem while awaiting results of tests for other pathogens. This was also effective in the treatment of syphilis. After all etiologic test results were obtained, the only positive result was for syphilis. However, SC associated with syphilis is rare, and the choice of broad-spectrum antibiotics was motivated by the safety and efficacy of treatment.

Tertiary syphilis occurs in one-third of patients with untreated syphilis. Half of them have late benign syphilis, a quarter have neurosyphilis, and a quarter exhibit cardiovascular syphilis [6]. It was estimated that cardiovascular syphilis accounted for 10–15% of clinical syphilis in the pre-penicillin era [7]. The most common complication of cardiovascular syphilis is syphilitic aortitis, which leads to aortic aneurysm (71%) in most cases, aortic insufficiency (47%), and coronary ostial stenosis (16.5%) in a minority of the patients [8]. Meanwhile, arteritis damages the vascular endothelium and activates the coagulation mechanism to cause coronary thrombosis, which may cause myocardial dysfunction and even myocardial infarction [9].

We excluded the diagnosis of conventional cardiovascular syphilis by considering the following points: (1) cardiovascular syphilis usually occurs 10–30 years after Treponema pallidum infection [8], while these patients were considered as having active early syphilis. (2) No evidence of aortic aneurysm and severe aortic insufficiency was found in either patient through chest CT scan, x-ray, or echocardiogram [10]. (3) In terms of prognosis, cardiovascular syphilis usually has significant complications but, in both cases, cardiac function and cardiac structures returned to normal within two weeks. It is possible that early syphilis also causes cardiomyopathy. On the other hand, we must acknowledge that the cardiovascular manifestations of syphilis are still dominated by traditional cardiovascular syphilis, such as aortitis, aneurysm, valvulitis, and coronary ostial stenosis, and that syphilis-associated septic cardiomyopathy may be an uncommon manifestation.

Further, the pathophysiological mechanism of septic cardiomyopathy is mitochondrial damage and oxidative stress [5]. Although Treponema pallidum lacks the structure of a typical lipopolysaccharide, it has abundant lipoproteins. These lipoproteins mediate inflammation by releasing monocyte phagolysosomes and generating many reactive oxygen species (ROS) [11]. The ROS impairs the mitochondrial oxidation respiratory chain, leading to further production of ROS. ROS generation by the mitochondria further stimulates endothelial cells to produce more ROS, establishing a vicious cycle [5]. ROS results in the destruction of DNA constant, and reduces the production of adenosine triphosphate, even inducing death of cells, including cardiomyocytes. In vivo or in vitro studies should be designed to illustrate the possible mechanism of syphilis-associated SC.

In conclusion, clinicians should consider Treponema pallidum in the case of septic cardiomyopathy with unknown pathogens. Meanwhile, when encountering patients with acute syphilis, clinicians should be alert to the development of SC. The specific pathophysiological mechanism of syphilis-associated septic cardiomyopathy has not been elucidated, and further clinical observation and laboratory research are needed.

## Data Availability

Not applicable.

## Abbreviations

BAL: Bronchoalveolar lavage
BNP: Brain natriuretic peptide
CRP: C-reactive protein
CRRT: Continuous renal replacement therapy
CT: Computed tomography
EF: Ejection fraction
ECMO: Extracorporeal membrane oxygenation
HIV: Human immunodeficiency virus
hs-cTnT: Hypersensitive troponin T
HR: Heart rate
IABP: Intra-aortic balloon pump
IMV: Invasive mechanical ventilation
Mb: Myoglobin
N%: Neutrophil percentage
NT-proBNP: N-terminal pro-brain natriuretic peptide
Ngs: Next-generation sequencing technology
ROS: Reactive oxygen species
RPR: Rapid plasma reagin test
SC: Septic cardiomyopathy
TRUST: Toluidine red unheated serum test
TPPA: Treponema pallidum particle agglutination
WBC: White blood cells
